# Effect of Drying Methods on Volatile Compounds of Burdock (*Arctium lappa* L.) Root Tea as Revealed by Gas Chromatography Mass Spectrometry-Based Metabolomics

**DOI:** 10.3390/foods10040868

**Published:** 2021-04-15

**Authors:** Junjie Xia, Zili Guo, Sheng Fang, Jinping Gu, Xianrui Liang

**Affiliations:** 1 Collaborative Innovation Center of Yangtze River Delta Region Green Pharmaceuticals, College of Pharmaceutical Sciences, Zhejiang University of Technology, Hangzhou 310014, China; xjj0718@163.com (J.X.); guozili@zjut.edu.cn (Z.G.); jinpinggu@foxmail.com (J.G.); 2 School of Food Science and Biotechnology, Zhejiang Gongshang University, Xuezheng Street No. 18, Hangzhou 310018, China; fszjgsu@163.com

**Keywords:** *Arctium lappa* L., drying methods, volatile compounds, HS-GC-MS, metabolomics

## Abstract

Burdock (*Arctium lappa* L.) is one of the nutritional foods widely planted in many countries. Dried burdock root (BR) is available as a herbal tincture and tea in many Asian countries with good flavor and taste. In this study, the volatile components in dried BR were identified and the effects of different drying methods on the volatile components were investigated by HS-GC-MS method. A total of 49 compounds were identified. Different drying methods including hot-air drying (HD, at 50, 60, 70, and 80 °C), vacuum drying (VD, at 50, 60, 70, and 80 °C), sunlight drying (SD), natural drying (ND), and vacuum freeze drying (VFD) were evaluated by HS-GC-MS-based metabolomics method. Results showed that different drying methods produced different effects on the volatile compounds. It was observed that 2,3-pentanedione, 1-(1H-pyrrol-2-yl)-ethanone, furfural, and heptanal were detected at higher concentrations in HD 80 and VD 70. The traditional HD and SD methods produced more flavor substances than VFD. The BR treated by the VFD method could maintain the shape of the fresh BR pieces while HD50 and VD80 methods could maintain the color of fresh BR pieces. These findings could help better understand the flavor of the corresponding processed BR and provide a guide for the drying and processing of BR tea.

## 1. Introduction

*Arctium lappa* L. (burdock), which is also called “Niubang” in China, is a biennial herb of the compositae family. It is not only a nutritious vegetable but also an important traditional Chinese medicine (TCM) that possesses antioxidant [1,2,3], antihyperglycemic [4], high fat diet-induced quail atherosclerosis protective effects [5], and other pharmacological effects [6]. It was reported that phenolic and polysaccharide were the main compounds [7,8]. In addition, many of the biological properties can be attributed to its phenolic compounds such as caffeoylquinic acids, chlorogenic acids, and their derivatives [9,10,11]. In addition to being consumed fresh or as a TCM ingredient, burdock root (BR) is primarily dried into tea in China and Southeast Asia. The aroma that is determined by a group of volatile organic compounds (VOCs) is an important factor of the quality of dried tea [12]. Drying is an essential step in the processing of dried burdock tea and the drying methods will have a great impact on the VOCs and flavor [13,14]. For example, high temperature drying and lyophilization caused the highest essential oil loss of *Thymus vulgaris* as analyzed by GC-MS [15]. Ye et al. [14] studied the effects of different drying methods on the aroma compounds of *Anoectochilus roxburghii* (Wall.) Lindl. and found that vacuum drying gave best results. Although studies about the separation and identification of nonvolatile compounds from BR have been performed, little is known about the VOCs in dried BR tea and the effects of different drying methods on it. 

Many methods have been developed and applied for the analysis of chemical components from burdock [11,16,17]. The composition of volatile components from burdock leaves has been determined by gas chromatography-mass spectrometry (GC-MS) [17]. In recent years, the GC-MS-based metabolomics method has been developed for the study of the aroma in food products and processing [18,19]. The GC-MS-based metabolomics method is very useful for comprehensive and systematic analysis of VOCs in biological samples, including alcohols, hydroxy acids, amino acids, fatty acids, sterols, etc. [20]. Xu et al. [21] applied an untargeted GC-MS-based plant metabolomics method for comparing volatile oil profiles of Mahuang and Mahuanggen. To the best of our knowledge, the GC-MS-based metabolomics approach has not yet been applied to analyze the effects of different drying methods of the VOCs in dried BR tea. 

In this work, the volatile compounds from the dried BR were investigated by headspace (HS)-GC-MS. Different drying treatments, including hot-air drying (HD, at 50, 60, 70, and 80 °C), sun drying (SD), vacuum freeze drying (VFD), vacuum drying (VD, at 50, 60, 70, and 80 °C), and natural drying (ND) were prepared and analyzed by the HS-GC-MS method. The multivariate statistical analysis including the orthogonal partial least squares discriminant analysis (OPLS-DA) and hierarchical cluster analysis (HCA) were applied to investigate the potential key discriminate compounds between ND and other drying methods.

## 2. Materials and Methods 

### 2.1. Materials

The fresh BR was collected from Linyi City, Shandong Province (35°05′ N, 118°24′ E) in September 2019, and these samples were identified based on morphological features by the lecturer Ai-cun Zhou from Zhejiang A & F University. The BR samples were stored in a refrigerator at 4 °C until use. These samples were rinsed and cut into small pieces (about 3 mm thick) before drying. The reference standards C_7_-C_40_ saturated n-alkane mixture (1000 mg/L) was purchased from Sigma-Aldrich Co., Ltd. (Shanghai, China).

### 2.2. Sample Preparation

#### 2.2.1. Hot-Air Drying (HD) Procedure 

About 60 g of freshly cut BR pieces were spread on the watch glasses, which were then placed in a drying oven (Shanghai Jinghong Experimental Equipment Co., Ltd., Shanghai, China). The samples were then dried to constant weight at 50, 60, 70, and 80 °C, respectively. 

#### 2.2.2. Sun Drying (SD) Procedure 

About 60 g of freshly cut BR pieces were spread on the watch glasses, which were put into a drug illuminate chamber (Shanghai Jianheng Instrument Co., Ltd., Shanghai, China). The samples were then dried to constant weight at 5900~6650 Lux.

#### 2.2.3. Vacuum Freeze-Drying (VFD) Procedure

About 60 g of freshly cut BR pieces were firstly frozen in an ultra-low temperature refrigerator (Thermo Scientific Forma 900 Series, American) at −80 °C for 4 h, and then were quickly placed into a freeze dryer with a cold-trap temperature of −80 °C and pressure of 3.4 Pa (FD-80, Beijing Boyikang Lab Instrument Co., Ltd., Beijing, China) for 48 h to achieve constant weight.

#### 2.2.4. Vacuum Drying (VD) Procedure

About 60 g of freshly cut BR pieces were spread on the watch glasses, which was put into a vacuum drying oven (DZF-6050, Shanghai Jinghong Experimental Equipment Co., Ltd., Shanghai, China). The samples were dried to constant weight at −0.01 MPa with temperature set at 50, 60, 70, and 80 °C, respectively.

#### 2.2.5. Natural Drying (ND) Procedure

About 60 g of freshly cut BR pieces were spread on the watch glasses and then were dried indoors to allow the moisture to evaporate naturally. The average room temperature and relative humidity were 20 ± 2 °C and 40%, respectively.

All the above samples for each drying method were prepared in triplicate. The biomass density of the BR pieces was around 0.36 g/cm^2^.

### 2.3. Color Measurement

CIELAB color space was used for the color characterization. The parameters L* (lightness), a* (balance between green and red), and b* (balance between yellow and blue) were measured by a Chroma Meter CR-400 colorimeter (Hangzhou Kesheng Instrument Co., Ltd., Hangzhou, China). Measurement was performed on random surface of the BR samples processed by different drying methods and the fresh one. Each group of samples was determined in triplicate and the values were expressed as mean ± SD. The total color differences (ΔE) were calculated by the following formula [22]:

(1) $$\sqrt{{\left(L_{0}^{*}-{\;L}^{*}\;\right)}^{2}+{\left(a_{0}^{*}-a^{*}\right)}^{2}}+{\left(b_{0}^{*}-b^{*}\right)}^{2}$$

where the L_0_*, a_0_*, and b_0_* referred to the parameters of fresh BR.

### 2.4. Water Activity (Aw) Determination

A dew point water activity meter analyzer (Aqualab 4TEV) was used to determine the water activity of all the dried BR samples processed by different drying methods. A random piece of dried BR sample was put into the water activity analyzer, and 20 min later, the readings were recorded.

### 2.5. Preparation of BR Powder

The dried BR pieces were ground into powder on a Retsch PM200 planetary ball mill (Retsch Inc., Haan, Germany). The ball milling parameters were: the rotating speed was 300 rpm and the rotating time was 15 min. The filling degree was 10.47%. The powder samples were kept in a refrigerator at 4 °C before analysis.

### 2.6. Preparation of QC Samples

The QC (quality control) samples were prepared by mixing equal amounts (1.15 g) of the BR powder obtained from different drying methods, and then analyzed in the same way as analytic samples [23,24].

### 2.7. HS-GC-MS Conditions

The HS-GC-MS analysis was carried out in an Agilent 7890B-5977B gas chromatograph-mass spectrometer equipped with an Agilent 7697A Headspace auto-sampler (Santa Clara, CA, USA). A J&W capillary column DB-624 UI (6% Cyanopropylphenyl, 94% dimethylsiloxane) of 30 m × 0.250 mm with 1.4 μm film (USA) was used for the separation. About 1.0 g of sample powder from each drying condition and QC samples were accurately weighed into a 20 mL sealed headspace vial and analyzed. Samples of each drying method were prepared in sextuplicate.

HS parameters were as follows: The BR samples in 20 mL headspace vial were heated at 120 °C with an equilibration time of 30 min. The temperatures of the headspace loop and the transfer line were maintained at 130 and 140 °C, respectively. The gas phase was injected into the GC-MS for analysis with the press equilibration time of 0.1 min and the injection time 1.0 min. A low shaker mode of the headspace vial was set up.

GC parameters were as follows: The carrier gas and make-up gas were high purity helium (>99.9%) and nitrogen (>99.9%), respectively. The carrier gas helium was used at a constant flow of 1.0 mL/min. The injector port was kept at 200 °C in split injection mode with split ratio 10:1. The oven temperature was ramped from 40 °C (held 5 min) to 170 °C at 8 °C/min, then to 230 °C (held 8 min) at 20 °C/min. The total run time was 32 min. All the samples along with QCs were injected into the GC-MS in a randomized order (QCs at regular intervals) to ensure the reliability of data. Additionally, the QC samples were injected at the beginning and at the end of the whole analytical run [21].

MS parameters were as follows: Data were acquired in electron impact (EI) mode at 70 eV, using the full scan mode over the mass range *m*/*z* 45 to 600 at 2.6 spectra/s. Temperatures of the ion source, the quadrupole, and the interface were set at 230, 150, and 250 °C, respectively. Data were analyzed by using Agilent MassHunter Qualitative Analysis (B.07.00). The identification of the VOCs was achieved by searching in the NIST14 spectral library by means of Automated Mass Spectral Deconvolution and Identification System (AMDIS) v2.72 as well as comparison of their GC retention time (RT), retention index (RI), and mass spectra with that of the saturated n-alkanes. The RI of the compound was calculated using C_7_-C_40_ n-alkanes, and the RT of the n-alkane was obtained with using the same HS-GC-MS conditions as BR samples.

### 2.8. Data Processing

The GC-MS raw data files were firstly converted into the Analysis Base File (ABF) format by AbfConverter (version 4.0.0) and then further processed by MS-DIAL (version 4.16) [25]. Data processing included data collection (mass scan range at 45–600 Da), peak detection, deconvolution, filtering (peak count filter was set as 8.1%, as there were 6 biological replicates and the total number of data was 74), peak alignment, and integration. An integrated data matrix composed of average RT, average RI, EI spectrum, sample name, and corresponding peak area was generated. Then, the peak area from the data matrix was normalized using the locally weighted scatterplot smoothing (LOWESS) method prior to further statistical analysis.

### 2.9. Statistical Analysis

The processed data was introduced into SIMCA 14.1 (Umetrics, Sweden) for multivariate statistical analyses, respectively. All variables were pareto-scaled before chemometric analysis [21]. The orthogonal partial least squares discriminant analysis (OPLS-DA) was utilized to study the differences between the SD, VFD, HD, VD, and ND samples. The differential volatile components were selected according to the variable importance in projection (VIP > 1.0) obtained from the OPLS-DA model and p-values (*p* < 0.05) calculated by Mann-Whitney U test. Meanwhile, fold changes (FC), calculated by the mean content of each compound, were applied to express the relative content changes of different treatments. The processed data were subject to log2 transformation. The hierarchical cluster analysis (HCA) with unsupervised method by R was used to reveal the aroma compound profiling changes among all the 11 samples of different drying methods. The heat map was obtained by using the *R*-4.0 language p heatmap package.

## 3. Results and Discussion

### 3.1. Appearance of Dried Burdock

**Figure 1:**
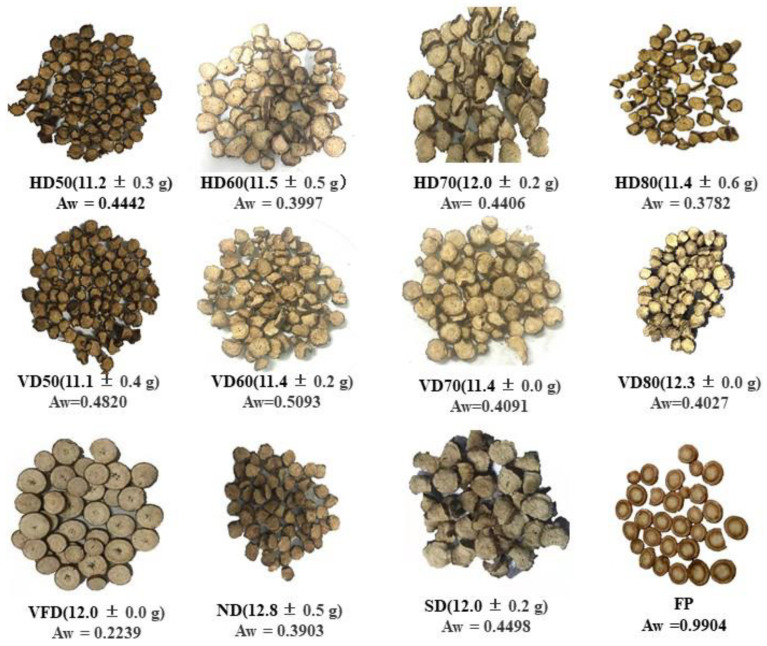
Appearance and dry weight of dried burdock root treated by different drying methods. (*n* = 3; the fresh weight for burdock is 60 g, and the dry weight are expressed as Mean ± SD; HD50–80 means hot air drying at 50–80 °C, VD50–80 means vacuum drying at 50–80 °C, VFD means vacuum freeze drying, ND means natural drying, SD means sun light drying, FP means fresh pieces and Aw means activity water).

The changes of dry weight for BR under different drying methods are shown in (Figure 1). The dry weight of burdock slices obtained under the 11 different drying methods ranged from 11.1 to 12.8 g (from 60 g fresh burdock) with little differences. So, the unbound water content in fresh BR that can be removed by drying is around 80.1 ± 0.4%. The result is in accordance with the moisture content of BR reported from the literature [26]. The water activity (Aw) of fresh BR is about 0.99, which is easy to spoil during storage. The Aw of BR under hot air drying, vacuum drying, vacuum freeze drying, natrual drying, and sun drying are 0.3782–0.4442, 0.4027–0.5093, 0.2239, 0.3903, and 0.4498, which are all below 0.6 [27]. Among them, the vacuum freeze-dried BR sample has the lowest water activity. 

The appearance of the dried burdock tea varied depending on the drying method used as shown in (Figure 1). The chroma data including the a*, b*, L*, and ΔE values were listed in (Table 1). The BR samples that underwent most drying methods were shrunken. However, the BR treated by the VFD method was white and with almost no shrinkage. VFD method could maintain the shape of fresh BR pieces. Compared with the fresh BR samples, no obvious decrease of L* values was observed except VD60. Besides, SD gave the highest L* value. The highest ΔE values were observed in the VD60 treated samples while the color of HD50 and VD80 methods were closest to that of fresh BR with the ΔE values at 4.68 and 4.82, respectively. Thus, these two drying methods were most able to maintain the color of fresh plants. 

### 3.2. Optimization of HS-GC-MS Parameters

The equilibrium temperature was the key factor affecting the response of volatile compounds in HS [28]. To obtain the optimal equilibrium temperature, different temperatures of 100, 110, 120, 130, and 140 °C were performed in HS analysis. With the increase of equilibrium temperature, the peak response will increase. However, the extreme high temperatures will lead to the degradation and transformation of thermally unstable compounds. Therefore, 120 °C was chosen as the preferred equilibrium temperature in this work.

Additionally, the column was also one of the vital conditions for chromatographic separation. Better chromatographic separation was a prerequisite for the analysis of compounds. In this study, two capillary columns including the HP-5 MS UI (5%-phenyl)-methyl polysiloxane of 30 m × 0.250 mm with 0.25 μm film (USA) and DB-624 UI (6% Cyanopropylphenyl, 94% dimethylsiloxane) of 30 m × 0.250 mm with 1.4 μm film (USA) were evaluated for their separating ability. The preferred resolution and better peak response were observed by using the DB-624 UI capillary column. 

**Figure 2:**
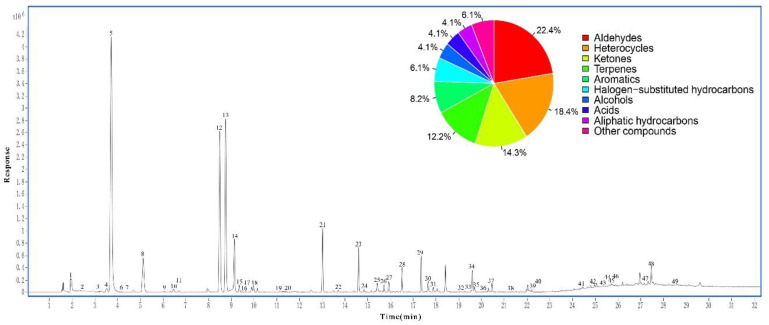
The total ion chromatogram (TIC) of burdock root (BR) sample.

Other HS-GC-MS conditions including the equilibrium time of HS, the injector port temperature and the oven temperature of GC, the ion source temperature and quadrupole temperature of MS, and so on, were also optimized based on the experimental data. The representative GC-MS total ion chromatogram (TIC) of BR under the optimized conditions is shown in (Figure 2). 

### 3.3. Volatile Compounds Characterization by HS-GC-MS

Volatile compounds were identified from the QC sample based on the average RT, calculated RI value, and mass spectrum obtained after data processing. To calculate the RI of the matched compounds, an n-alkane mixture was run after the sample sequence. The identification criteria of a compound was the match score in the NIST greater than 750, or the difference between the calculated RI and that given from the NIST14 library was less than 50. RI was widely applied to identify compounds. Some structurally similar compounds with similar mass spectra can be distinguished due to their different RI [29]. The formulae for calculating RI value was as follows:

(2) $$\mathrm{RI}=100Z+100\;\times \;\frac{\mathrm{TR}\left(x\right)-\mathrm{TR}\left(z\right)}{\mathrm{TR}\left(z+1\right)-\mathrm{TR}\left(z\right)}$$

where TR(x), TR(z), and TR(z+1) represent the retention time of the component, the n-alkane with carbon number at z and z + 1, respectively, and TR(z) < TR(x) < TR(z + 1). 

In the study, a total of 49 volatile compounds were identified from BR (Table 2). These compounds mainly comprised of 11 aldehydes, 7 ketones, 9 heterocycles, 6 terpenes, 4 aromatics, 3 halogen-substituted hydrocarbons, 2 alcohols, 2 acids, 2 aliphatic hydrocarbons, and 3 other compounds. 

Aldehydes were one of the main volatile components in the dried BR. Compounds 4, 10, 12, 13, 17, 21, 23, 26, 31, 36, and 39 were identified as aldehydes. It is known that aldehydes are the main components of essential oils from many plants which have high antimicrobial activities and valuable applications [30,31,32]. Among them, 3-methylbutanal (compound 12), 2-methylbutanal (compound 13), hexanal (compound 21), and furfural (compound 23) were found to be the main components. Take compound 21 as an example to explain the identification work of these aldehydes. The mass spectrum matching result showed that hexanal had a high matching score of 954. The calculated RI of compound 21 was 830, which was similar with the database retention index of 800. As a result, compound 21 was identified as hexanal derived from oxidative decomposition of fatty acids. Thermal oxidation of cholesterol and Maillard reaction can lead to development of 2-methylbutanal and 3-methylbutanal. Furfural also has a role as a Maillard reaction product and a metabolite. It is a member of furans and an aldehyde which can derive from furan. 

Compounds 9, 15, 22, 24, 27, 29, 33, 38, and 40 were identified as heterocycles. Compound 29 with relatively high content in the dried BR was used as an example to elucidate the identification work of these heterocycles. The mass spectrum matching result showed that 2-pentylfuran had a high matching score of 951. The calculated RI of compound 29 was 1007, which was similar with the library RI of 993 and thus the compound 29 was identified as 2-pentylfuran.

### 3.4. Comparisons of SD, VFD, HD, VD, and ND on the Volatile Compounds of BR by OPLS-DA

To further explore the differential compounds among the different drying methods, the OPLS-DA for pairwise comparison was carried out. Table 3 listed the overall view of FC values between different drying methods and ND. More volatile substances were observed in HD drying methods than in VD due to the oxidization or degradation of components at high temperatures. For HD80, 2,3-Pentanedione was found to be in high content. Formic acid and 2-ethylfuran were observed in all the groups. It can be seen that compared with ND, the proportion of formic acid in the other drying methods has all increased. However, the 2-ethylfuran decreased as the FC values were below 1. Besides, the hexanal, 2-heptanone, and 2-pentylfuran also decreased in most of the drying methods. The smallest FC values were observed in VFD. This result was in accordance with the findings for volatiles after VFD in coffee beans [33] and Chinese ginger [34] that VFD could not produce volatile flavor substances from samples. The volatile compounds that did not meet the identification criterion were noted as unknown.

#### 3.4.1. Comparison between SD and ND

**Figure 3:**
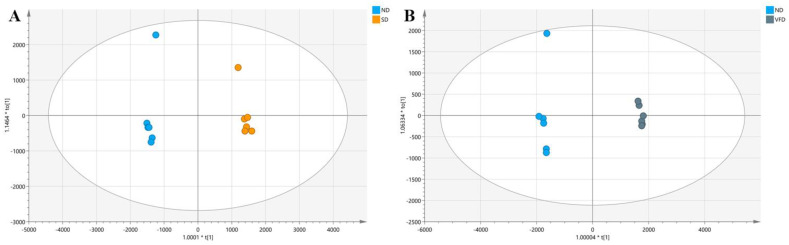
The OPLS−DA scores plot: (**A**) comparison between natural drying (ND) and sunlight drying (SD), (**B**) comparison between ND and vacuum freeze drying (VFD); (*n* = 6).

The different effects on VOCs of BR between SD and ND were displayed in (Figure 3A) and the corresponding loading plots are shown in (Figure S1A). Table S1 lists the main 19 volatile components identified based on VIP > 1 and *p*-value < 0.05. Among them, dimethyl sulfide had the highest VIP value, followed by 2-methylbutanal and 3-methylbutanal. The relative contents of aldehydes (3-methylbutanal, 2-methylbutanal, pentanal, propanal, and furfural) and terpenes (β-elemene, β-Selinene, and α-Selinene) were observed more in SD than ND. Terpenes are a series of bioactive compounds which have anticancer [35,36] and antifungal [37,38] activities. The terpenes identified in this work were all sesquiterpenes (C15) that could be synthesized through the MVA pathway [39]. Another six differential heterocyclic compounds were found to be the important components of plant flavor and the main components of heated-treated food, especially pyrazines. Besides, formic acid, chloromethane, and dimethyl sulfide were also the potential chemical markers for differentiating SD from ND.

#### 3.4.2. Comparison between VFD and ND

VFD was considered as the forefront of drying research as it could maintain the shape of the final products [40] as well as good quality and nutrition of food [41], however, it often needs high energy consumption and cannot preserve the aroma substances. 

Separation between VFD and ND was shown in (Figure 3B) with high value of R2Y and Q2. As shown in (Table S2), the relative percentage of aldehydes exhibited more in VFD than in ND. Besides, the differential aliphatic hydrocarbons and two ketones (2-butanone and 2-heptanone) between VFD and ND were also observed. Aliphatic hydrocarbon compounds are derived from oxidative decomposition of lipids [42]. In this research, three differential aliphatic hydrocarbons were identified, 1-pentadecene, heptadeca-1,8,11-triene, and heptadeca-1,8,11,14-tetraene. They have relatively high odor threshold values and usually lack substantial impact on flavor [43]. Besides, another differential compound with VIP 1.59 was identified as the terpene1-methyl-4-(6-methylhept-5-en-2-yl) benzene, which has the antimicrobial and antioxidant activities [44]. The loading plots between VFD and ND are shown in Figure S1B.

#### 3.4.3. Comparison between HD and ND

HD is one of the most conventional drying processes widely used in the food industry [45] with the advantages of easy-operation and low cost [46]. However, it will also take away part of the volatile substances while performing the drying process. The effect of temperature on food is ultimately determined by its effect on chemical components as high temperature usually results in the degradation and loss of important flavor substances. 

**Figure 4:**
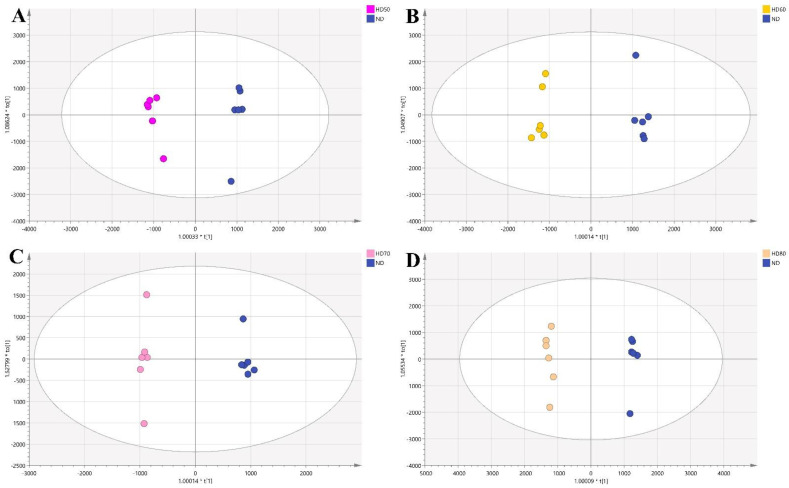
The OPLS−-DA scores plot for comparisons between the different temperatures of hot air drying (HD) and natural drying (ND) (*n* = 6): (**A**) for HD50 and ND, (**B**) for HD60 and ND, (**C**) for HD70 and ND, (**D**) for HD80 and ND.

In this work, comparisons of HD (named HD50, HD60, HD70, and HD80 at 50, 60, 70, and 80 °C, respectively) and ND were performed. Four OPLS-DA models with distinct separations are shown in Figure 4. All the models showed good fitness and strong predictability with high value of R2Y and Q2. Afterwards, the key differential compounds of four sets of comparison selected according to VIP > 1 and *p* < 0.05 were displayed in (Tables S3–S6), respectively. The labels of ID were exported after peak alignment by MS-DIAL. Compounds that did not meet the identification criteria were recorded as unknown. A total of 27, 21, 25, and 30 compounds were screened from the four groups HD50-ND, HD60-ND, HD70-ND, and HD80-ND, respectively. The corresponding loading plots are shown in (Figure S2).

#### 3.4.4. Comparison between VD and ND

VD samples dried under negative pressure, reducing the processing time and allowing less oxygen exposure time [47]. Different temperatures of VD could have different effects. High temperature may lead to the degradation of biologically active components in the plant.

**Figure 5:**
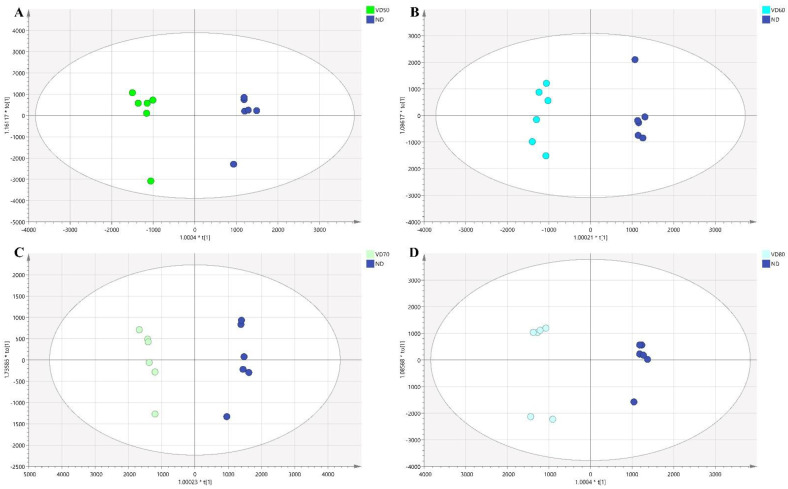
The OPLS−DA scores plot for comparisons between the different temperatures of vacuum drying (VD) and natural drying (ND) (*n* = 6): (**A**) for VD50 and ND, (**B**) for VD60 and ND, (**C**) for VD70 and ND, and (**D**) for VD80 and ND.

The supervised OPLS-DA was performed to understand the volatile compounds contributing to differentiation. In the OPLS-DA score plots (Figure 5), a group of clear discrimination was observed between different temperatures of VD (named VD50, VD60, VD70, and VD80 at 50, 60, 70, and 80 °C, respectively). The models were stable and reproducible. A total of 21, 12, 9, and 14 compounds in VD50, VD60, VD70, and VD80 groups were selected and identified based on the criteria of VIP > 1 and *p* < 0.05 (Tables S7–S10), respectively. The corresponding loading plots are shown in (Figure S3).

### 3.5. HCA of Volatile Compounds in BR

**Figure 6:**
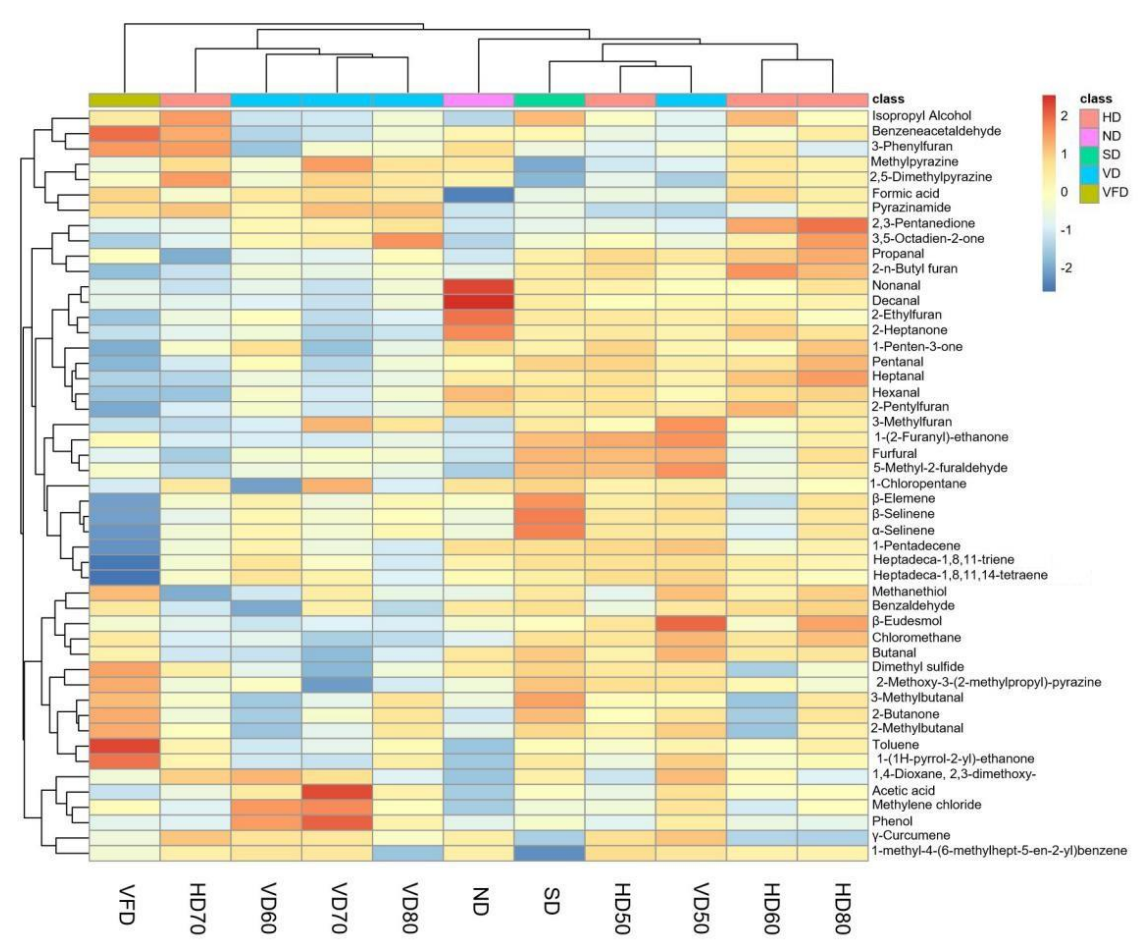
Heatmap of the volatile profiles of burdock root (BR). The legend on the left and bottom indicates the grouping of volatiles and sample groups, respectively (HD: hot air drying, ND: natural drying, SD: sunlight drying, VD: vacuum drying, VFD: vacuum freeze drying) (*n* = 6).

To further compare the contents of volatiles and assess the correlation among the different drying methods, a heatmap was obtained after hierarchical cluster analysis (HCA) of the volatiles of the BR as shown in (Figure 6). The content value of each volatile was normalized by log2 transformation. The analysis was unsupervised. The Euclidean distance was used in the cluster analysis to display similarity among sample groups. The shorter the Euclidean distance, the greater similarities of the samples are. Similar squares were jointed together as they formed into cluster. Two major clusters were produced. The VFD group was in one cluster while all the other groups were in another cluster, suggesting the significant differences of VFD from others. Some of the key distinctly aroma volatile compounds were propanal, 2-pentylfuran, 1-(2-furanyl)-ethanone, nonanal, pentanal, and furfural, which were abundant in the HD, VD, and SD group, and less in the VFD group. HD50, HD60, and HD80 were in the same subclass while VD60, VD70, and VD80 were in other subclass. The effects of these temperatures were similar from HCA. SD was close to HD50 and VD50.

## 4. Conclusions

In the current study, HS-GC-MS was used to analyze the volatile components in BR. Different drying methods including HD and VD at different temperatures, ND, SD, and VFD were investigated for their influence on the volatile compounds of BR by using the HS-GC-MS coupled with multivariate statistical analysis. The main volatile components were aldehydes, ketones, heterocycles, terpenes, aromatics, and so on. Different drying methods exerted different influences on types and the content of volatile compounds of BR. All the OPLS-DA models showed distinct separations, good interpretation, and predictability between the different groups. Results revealed that the most remarkable representative differential volatiles were dimethyl sulfide, 2-ethylfuran, furfural, and aldehydes. HCA suggested that the VFD was the most different drying procedure. The traditional drying methods (HD and SD) could be used to produce flavor substances as oxidation, Maillard reactions, and degradation reactions may take place during the drying process. The results obtained in this work provide an overall profile for understanding the volatile compounds in BR and also provide a guide for the drying, storing, and processing of BR.

## Figures and Tables

**Table 1:** Effects of drying methods on Chroma value of dried burdock root.

Treatment ^1^	a*	b*	L*	ΔE
HD50	3.45 ± 0.23	23.41 ± 0.42	70.34 ± 0.73	4.68
HD60	2.01 ± 1.02	24.87 ± 1.24	71.87 ± 0.93	6.89
HD70	2.70 ± 0.47	23.12 ± 0.57	64.37 ± 1.04	4.97
HD80	2.73 ± 0.22	26.22 ± 1.07	72.86 ± 0.59	7.55
VD50	4.05 ± 0.37	17.39 ± 0.95	62.34 ± 0.90	8.27
VD60	2.78 ± 0.32	17.75 ± 0.58	53.70 ± 1.33	15.16
VD70	3.63 ± 0.14	14.16 ± 1.59	64.02 ± 1.40	10.40
VD80	4.06 ± 1.54	20.77 ± 2.71	64.32 ± 1.56	4.82
ND	2.40 ± 1.44	20.05 ± 4.54	66.06 ± 0.43	5.74
SD	1.96 ± 0.24	26.35 ± 0.38	76.56 ± 1.02	10.99
VFD	2.75 ± 0.73	16.76 ± 2.83	66.86 ± 0.51	7.93
FP	6.83 ± 0.61	23.56 ± 0.48	67.11 ± 0.87	/

^1^ HD50–80: hot air drying at 50–80 °C; VD50–80: vacuum drying at 50–80 °C; ND: natural drying; SD: sun light drying; VFD: vacuum freeze drying; FP: fresh pieces.

**Table 2:** A total of 49 volatile compounds were identified from burdock root ^1^.

No.	Compounds	CAS	RT/min	Formula	Mw	Score	RIt	RIc
1	Chloromethane	74-87-3	1.911	CH_3_Cl	50.49	992	329	484.2
2	Methanethiol	74-93-1	2.448	CH_4_S	48.11	930	401	497.0
3	Formic acid	64-18-6	3.116	C_2_H_6_O	46.07	803	526	522.7
4	Propanal	123-38-6	3.439	C_3_H_6_O	58.08	986	461	532.5
5	Dimethyl sulfide	75-18-3	3.653	C_2_H_6_S	62.13	937	520	539.2
6	Isopropyl Alcohol	67-63-0	3.846	C_3_H_8_O	60.10	974	486	545.7
7	Methylene chloride	75-09-2	4.194	CH_2_Cl_2_	84.93	862	528	556.4
8	2-Butanone	78-93-3	4.998	C_4_H_8_O	72.11	814	598	581.7
9	3-Methylfuran	930-27-8	5.97	C_5_H_6_O	82.10	940	614	612.2
10	Butanal	123-72-8	6.56	C_4_H_8_O	72.11	855	593	631.0
11	2,3-Dimethoxy-1,4-dioxane	23918-30-1	6.747	C_6_H_12_O_4_	148.16	731	/	636.7
12	3-Methylbutanal	590-86-3	8.36	C_5_H_10_O	86.13	934	652	687.6
13	2-Methylbutanal	96-17-3	8.622	C_5_H_10_O	86.13	939	662	695.8
14	Acetic acid	64-19-7	9.164	C_2_H_4_O	60.05	984	610	712.8
15	2-Ethylfuran	3208-16-0	9.273	C_6_H_8_O	96.13	949	703	716.4
16	1-Penten-3-one	1629-58-9	9.52	C_5_H_8_O	84.12	841	681	724.2
17	Pentanal	110-62-3	9.775	C_5_H_10_O	86.13	871	699	732.2
18	2,3-Pentanedione	600-14-6	9.974	C_5_H_8_O_2_	100.12	904	698	734.5
19	1-Chloropentane	543-59-9	11.043	C_5_H_11_Cl	106.59	891	742	772.2
20	Toluene	108-88-3	11.539	C_7_H_8_	92.14	831	763	787.8
21	Hexanal	66-25-1	12.893	C_6_H_12_O	100.16	952	800	830.6
22	Methylpyrazine	109-08-0	13.608	C_5_H_6_N_2_	94.11	827	831	861.1
23	Furfural	98-01-1	14.477	C_5_H_4_O_2_	96.08	959	833	892.6
24	2-n-Butyl furan	4466-24-4	14.851	C_8_H_12_O	124.18	850	893	907.0
25	2-Heptanone	110-43-0	15.411	C_7_H_14_O	114.19	781	891	930.1
26	Heptanal	111-71-7	15.598	C_7_H_14_O	114.19	860	901	937.7
27	2,5-Dimethylpyrazine	123-32-0	15.818	C_6_H_8_N_2_	108.14	885	917	946.8
28	1-(2-Furanyl)-ethanone	1192-62-7	16.382	C_6_H_6_O_2_	110.11	964	911	970.0
29	2-Pentylfuran	3777-69-3	17.267	C_9_H_14_O	138.21	956	993	1007.1
30	Benzaldehyde	100-52-7	17.542	C_7_H_6_O	106.12	952	962	1019.6
31	5-Methyl-2-furaldehyde	620-02-0	17.768	C_6_H_6_O_2_	110.11	907	980	1029.9
32	Phenol	108-95-2	19.193	C_6_H_6_O	94.11	905	980	993.9
33	Pyrazinamide	98-96-4	19.403	C_5_H_5_N_3_O	123.11	844	1250	1104.7
34	Benzeneacetaldehyde	122-78-1	19.472	C_8_H_8_O	120.15	942	1051	1108.1
35	3,5-Octadien-2-one	38284-27-4	19.83	C_8_H_12_O	124.18	705	1091	1125.6
36	Nonanal	124-19-6	20.176	C_9_H_18_O	142.24	767	1104	1142.7
37	1-(1H-pyrrol-2-yl)-ethanone	1072-83-9	20.356	C_6_H_7_NO	109.13	852	1072	1151.8
38	2-Methoxy-3-(2-methylpropyl)-pyrazine	24683-00-9	21.41	C_9_H_14_N_2_O	166.22	871	1183	1205.2
39	Decanal	112-31-2	22.1	C_10_H_20_O	156.27	772	1206	1249.0
40	3-Phenylfuran	13679-41-9	22.637	C_10_H_8_O	144.17	845	1226	1283.0
41	β-Elemene	515-13-9	24.375	C_15_H_24_	204.35	910	1391	1431.7
42	1-Pentadecene	13360-61-7	24.975	C_15_H_30_	210.40	915	1492	1491.1
43	γ-Curcumene	451-55-8	25.172	C_15_H_24_	204.35	814	1480	1509.6
44	1-Methyl-4-(6-methylhept-5-en-2-yl) benzene	644-30-4	25.251	C_15_H_22_	202.34	936	1483	1516.7
45	β-Selinene	17066-67-0	25.613	C_15_H_24_	204.35	870	1486	1548.9
46	α-Selinene	473-13-2	25.67	C_15_H_24_	204.35	767	1494	1554.0
47	Heptadeca-1,8,11-triene	56134-03-3	27.194	C_17_H_30_	234.42	865	1665	1675.7
48	Heptadeca-1,8,11,14-tetraene	10482-53-8	27.333	C_17_H_28_	232.40	955	1664	1686.1
49	β-Eudesmol	473-15-4	28.444	C_15_H_26_O	222.37	819	1649	1754.9

^1^ CAS: CAS Registry Number; RT: retention time; Mw: molecular weight; RIt: the theoretical retention index in the NIST14 library; RIc: the calculated retention index.

**Table 3:** The overall view of fold-changes (FC) values between different drying methods and natural drying (ND) ^1^.

RT/min	Compound	HD50/ND	HD60/ND	HD70/ND	HD80/ND	SD/ND	VD50/ND	VD60/ND	VD70/ND	VD80/ND	VFD/ND
1.911	Chloromethane	2.52	2.54	/	3.50	2.73	3.72	/	/	0.76	2.33
2.318	Methanethiol	/	/	/	1.83	/	/	/	/	/	/
3.115	Unknown	2.76	6.23	3.33	4.82	2.65	2.76	5.30	6.02	5.35	6.47
3.116	Formic acid	2.83	6.40	3.40	4.94	2.79	2.83	5.46	6.22	5.51	6.65
3.439	Propanal	1.39	1.42	/	1.51	1.28	1.32	/	/	/	/
3.557	Unknown	/	/	/	0.70	/	/	0.62	/	0.62	1.65
3.625	Unknown	/	/	/	0.65	1.24	/	0.57	/	0.60	1.66
3.653	Dimethyl sulfide	/	0.34	/	0.65	1.32	/	0.52	/	0.61	1.75
3.846	Isopropyl alcohol	/	12.12	17.35	/	12.68	/	/	/	/	/
4.194	Methylene chloride	/	/	/	/	/	/	28.90	33.94	/	/
4.595	Unknown	2.55	2.66	/	3.45	4.18	3.14	/	/	/	2.48
4.998	2-Butanone	1.30	/	/	1.52	1.76	1.53	/	/	1.53	1.85
6.219	Unknown	0.27	0.22	0.10	0.21	0.35	0.21	0.15	0.07	0.10	0.09
6.327	Unknown	/	/	/	/	/	1.67	/	/	/	/
8.360	3-Methylbutanal	/	0.71	/	1.36	1.75	/	/	/	/	1.63
8.622	2-Methylbutanal	1.32	0.75	/	1.33	1.56	1.60	/	/	/	1.78
9.164	Acetic acid	/	1.57	/	1.61	1.57	2.08	1.90	/	/	/
9.273	2-Ethylfuran	0.42	0.45	0.20	0.26	0.40	0.35	0.28	0.13	0.16	0.10
9.775	Pentanal	1.41	/	/	1.57	1.41	/	/	/	/	0.50
9.974	2,3-Pentanedione	/	/	/	21.39	/	/	/	/	/	/
9.996	Unknown	/	3.49	3.82	/	/	/	/	3.61	/	/
11.043	1-Chloropentane	2.94	2.55	/	3.06	/	2.55	/	/	/	/
11.190	Unknown	2.36	3.12	/	3.90	/	4.79	4.22	/	/	/
11.311	Unknown	/	2.61	/	/	/	/	/	/	/	/
12.481	Unknown	/	0.26	/	/	2.79	/	/	/	/	/
12.893	Hexanal	/	/	0.22	/	/	0.55	0.47	0.31	0.45	0.22
13.608	Methylpyrazine	0.50	/	/	/	0.34	0.54	/	/	/	/
14.477	Furfural	3.66	/	/	2.85	3.77	3.85	1.55	/	/	/
14.666	Unknown	/	0.16	0.16	0.10	/	0.20	/	0.06	0.08	/
15.081	Unknown	/	/	0.61	/	/	/	/	/	/	/
15.411	2-Heptanone	0.57	/	0.32	/	/	0.51	0.37	0.23	0.27	0.25
15.818	2,5-Dimethylpyrazine	0.67	/	1.68	/	0.42	0.49	/	1.32	/	/
16.382	1-(2-Furanyl)-ethanone	4.74	/	/	2.46	4.08	5.47	/	/	/	2.04
16.722	Unknown	0.28	0.18	0.30	/	3.80	/	/	0.88	/	/
17.267	2-Pentylfuran	/	/	0.37	/	/	/	0.54	0.35	0.45	0.21
17.542	Benzaldehyde	/	/	0.18	/	/	/	0.67	/	/	/
17.768	5-Methyl-2-furancarboxaldehyde	4.05	/	/	2.86	4.19	5.26	/	/	/	/
17.948	Unknown	/	/	1.84	/	/	/	/	/	/	/
19.074	Unknown	/	/	/	5.28	/	/	/	/	/	/
19.193	Phenol	/	/	/	/	/	/	62.08	10.54	/	/
19.403	Pyrazinamide	/	/	2.42	/	/	/	/	/	2.48	/
19.472	Benzeneacetaldehyde	/	/	/	/	/	/	0.72	/	/	1.46
19.509	Unknown	/	/	2.84	/	/	/	/	/	/	/
19.830	3,5-Octadien-2-one	/	/	/	5.08	/	/	/	/	5.34	/
20.110	Unknown	/	/	/	/	3.72	/	/	/	/	/
20.176	Nonanal	/	/	0.27	/	/	/	/	/	/	/
20.356	1-(1H-pyrrol-2-yl)-ethanone	/	/	1.93	/	2.21	2.61	/	/	2.10	3.75
21.194	Unknown	3.41	/	/	/	/	/	/	/	/	/
21.410	2-Methoxy-3-(2-methylpropyl)-pyrazine	2.95	/	/	/	3.89	2.78	/	/	/	4.81
21.973	Unknown	/	/	/	12.22	/	/	/	/	10.39	/
21.977	Unknown	/	/	4.66	22.67	/	6.19	7.59	11.92	13.95	10.52
22.100	Decanal	/	/	0.25	/	/	/	/	/	/	/
22.761	Unknown	/	/	/	/	/	/	42.63	/	/	/
23.031	Unknown	/	/	/	/	/	1.84	/	/	/	/
24.375	β-Elemene	/	/	/	1.71	3.25	1.83	/	/	/	/
24.616	Unknown	/	/	/	/	/	/	0.24	/	/	/
24.881	Unknown	/	/	/	/	/	/	/	/	/	6.38
24.975	1-Pentadecene	/	0.65	0.63	/	/	/	/	0.61	0.50	0.31
25.172	γ-Curcumene	/	/	1.77	/	/	/	/	/	/	/
25.251	1-Methyl-4-(6-methylhept-5-en-2-yl) benzene	1.44	/	/	/	0.12	/	/	/	0.21	0.54
25.316	Unknown	/	/	/	/	3.94	/	/	/	/	/
25.361	Unknown	/	/	/	/	/	29.56	/	/	7.37	/
25.547	Unknown	2.40	/	/	/	/	/	/	/	/	/
25.613	β-Selinene	1.84	/	/	/	3.89	2.11	/	/	/	/
25.670	α-Selinene	1.87	/	/	1.96	3.78	1.90	/	/	/	/
26.828	Unknown	/	2.66	/	/	3.31	2.15	/	/	/	28.47
27.194	Heptadeca-1,8,11-triene	/	/	/	/	/	/	/	/	0.63	0.36
27.333	Heptadeca-1,8,11,14-tetraene	1.26	/	/	/	/	/	/	/	/	0.41
27.418	Unknown	2.10	/	/	/	/	/	/	/	/	/
27.521	Unknown	/	/	7.27	/	/	/	/	/	/	/
27.577	Unknown	/	/	/	/	/	/	/	/	4.41	/
28.444	β-Eudesmol	/	/	/	13.25	/	31.16	/	/	/	/
28.517	Unknown	/	/	/	/	/	11.25	/	/	/	/
28.903	Unknown	/	/	2.24	/	/	/	2.25	/	2.14	2.80
29.377	Unknown	/	/	/	/	/	/	/	/	/	8.06

^1^ Volatile compounds that did not meet the identification criterion were noted as unknown. HD50–80: hot air drying at 50–80 °C; VD50–80: vacuum drying at 50–80 °C; ND: natural drying; SD: sun light drying; VFD: vacuum freeze drying.

## Data Availability

Data sharing is not applicable to this article.

## Supplementary Materials

The following are available online at https://www.mdpi.com/article/10.3390/foods10040868/s1, Table S1: The main 31 volatile components identified based on VIP > 1 and *p*-value < 0.05 between sun light drying(SD) and natural drying (ND), Table S2: The main 29 volatile components identified based on VIP > 1 and *p*-value < 0.05 between vacuum freeze drying (VFD) and natural drying (ND), Table S3: The main 27 volatile components identified based on VIP > 1 and *p*-value < 0.05 between hot air drying at 50 °C (HD50) and natural drying (ND), Table S4: The main 21 volatile components identified based on VIP > 1 and *p*-value < 0.05 between hot air drying at 60 °C (HD60) and natural drying (ND), Table S5: The main 25 volatile components identified based on VIP > 1 and *p*-value < 0.05 between hot air drying at 70 °C (HD70) and natural drying (ND), Table S6: The main 30 volatile components identified based on VIP > 1 and *p*-value < 0.05 between hot air drying at 80 °C (HD80) and natural drying (ND), Table S7: The main 32 volatile components identified based on VIP > 1 and *p*-value < 0.05 between vacuum drying at 50 °C (VD50) and natural drying (ND), Table S8: The main 21 volatile components identified based on VIP > 1 and *p*-value < 0.05 between vacuum drying at 60 °C (VD60) and natural drying (ND), Table S9: The main 15 volatile components identified based on VIP > 1 and *p*-value < 0.05 between vacuum drying at 70 °C (VD70) and natural drying (ND), Table S10: The main 24 volatile components identified based on VIP > 1 and *p*-value < 0.05 between vacuum drying at 80 °C (VD80) and natural drying (ND). Figure S1: The loading plots: (A) between natural drying (ND) and sun light drying (SD), (B) between ND and vacuum freeze drying (VFD); Figure S2: The loading plots for comparisons between the different temperatures of hot air drying (HD) and natural drying (ND): A for HD50 and ND, B for HD60 and ND, C for HD70 and ND, D for HD80 and ND; Figure S3: The loading plots for comparisons between the different temperatures of vacuum drying (VD) and natural drying (ND): A for VD50 and ND, B for VD60 and ND, C for VD70 and ND, D for VD80 and ND.

## References

[1] Liu W., Wang J., Zhang Z., Xu J., Xie Z., Slavin M., Gao X. (2014). In vitro and in vivo antioxidant activity of a fructan from the roots of *Arctium lappa* L.. Int. J. Biol. Macromol..

[2] Maghsoumi-Norouzabad L., Shishehbor F., Abed R., Zare Javid A., Eftekhar-Sadat B., Alipour B. (2019). Effect of *Arctium lappa* linne (Burdock) root tea consumption on lipid profile and blood pressure in patients with knee osteoarthritis. J. Herbal Med..

[3] Predes F.S., Ruiz A.L., Carvalho J.E., Foglio M.A., Dolder H. (2011). Antioxidative and in vitro antiproliferative activity of *Arctium lappa* root extracts. BMC Complement. Altern. Med..

[4] Tousch D., Bidel L.P., Cazals G., Ferrare K., Leroy J., Faucanie M., Chevassus H., Tournier M., Lajoix A.D., Azay-Milhau J. (2014). Chemical analysis and antihyperglycemic activity of an original extract from burdock root (*Arctium lappa*). J. Agric. Food Chem..

[5] Wang Z., Li P., Wang C., Jiang Q., Zhang L., Cao Y., Zhong W., Wang C. (2016). Protective effects of *Arctium lappa* L. root extracts (AREs) on high fat diet induced quail atherosclerosis. BMC Complement. Altern. Med..

[6] Chan Y.S., Cheng L.N., Wu J.H., Chan E., Kwan Y.W., Lee S.M., Leung G.P., Yu P.H., Chan S.W. (2011). A review of the pharmacological effects of *Arctium lappa* (burdock). Inflammopharmacology.

[7] Lin L.-Z., Haenly J.M. (2008). Identification of Hydroxycinnamoylquinic Acids of arnica flowers and burdock roots using a standardized LC-DAD-ESI/MS profiling method. J. Agric. Food Chem..

[8] Wang Y., Zhang N., Kan J., Zhang X., Wu X., Sun R., Tang S., Liu J., Qian C., Jin C. (2019). Structural characterization of water-soluble polysaccharide from *Arctium lappa* and its effects on colitis mice. Carbohydr. Polym..

[9] Zheng Z., Wang X., Liu P., Li M., Dong H., Qiao X. (2018). Semi-preparative separation of 10 caffeoylquinic acid derivatives using high speed counter-current chromatogaphy combined with semi-preparative HPLC from the roots of burdock (*Arctium lappa* L.). Molecules.

[10] Jiang X.-W., Bai J.-P., Zhang Q., Hu X.-L., Tian X., Zhu J., Liu J., Meng W.-H., Zhao Q.-C. (2016). Caffeoylquinic acid derivatives from the roots of *Arctium lappa* L. (burdock) and their structure–activity relationships (SARs) of free radical scavenging activities. Phytochem. Lett..

[11] Jaiswal R., Kuhnert N. (2011). Identification and characterization of five new classes of chlorogenic acids in burdock (*Arctium lappa* L.) roots by liquid chromatography/tandem mass spectrometry. Food Funct..

[12] Du L.L., Fu Q.Y., Xiang L.P., Zheng X.Q., Lu J.L., Ye J.H., Li Q.S., Polito C.A., Liang Y.R. (2016). Tea polysaccharides and their bioactivities. Molecules.

[13] Guimarães R.M., Ida E.I., Falcão H.G., de Rezende T.A.M., de Santana Silva J., Fernandes Alves C.C., da Silva Pereira M.A., Egea M.B. (2020). Evaluating technological quality of okara flours obtained by different drying processes. LWT-Food Sci. Technol..

[14] Ye S., Wang Z., Shen J., Shao Q., Fang H., Zheng B., Younis A. (2019). Sensory qualities, aroma components, and bioactive compounds of *Anoectochilus roxburghii* (Wall.) Lindl. as affected by different drying methods. Ind. Crops Prod..

[15] Sárosi S., Sipos L., Kókai Z., Pluhár Z., Szilvássy B., Novák I. (2013). Effect of different drying techniques on the aroma profile of Thymus vulgaris analyzed by GC–MS and sensory profile methods. Ind. Crops Prod..

[16] Ferracane R., Graziani G., Gallo M., Fogliano V., Ritieni A. (2010). Metabolic profile of the bioactive compounds of burdock (*Arctium lappa*) seeds, roots and leaves. J. Pharm. Biomed. Anal..

[17] Kopyt’ko Y.F., Kir’yanov A.A., Stikhin Y.V., Stikhin V.A., Sokol’skaya T.A. (2003). Composition of volatile substances and fatty acids isolated from the juice of woolly burdock leaves. Pharm. Chem. J..

[18] Diez-Simon C., Mumm R., Hall R.D. (2019). Mass spectrometry-based metabolomics of volatiles as a new tool for understanding aroma and flavour chemistry in processed food products. Metabolomics.

[19] Li X., Tsuta M., Hayakawa F., Nakano Y., Kazami Y., Ikehata A. (2021). Estimating the sensory qualities of tomatoes using visible and near-infrared spectroscopy and interpretation based on gas chromatography-mass spectrometry metabolomics. Food Chem..

[20] Khodadadi M., Pourfarzam M. (2020). A review of strategies for untargeted urinary metabolomic analysis using gas chromatography-mass spectrometry. Metabolomics.

[21] Lv M., Sun J., Min W., Fan H., Zunjian Z., Fengguo X. (2016). Comparative analysis of volatile oils in the stems and roots of Ephedra sinica via GC-MS-based plant metabolomics. Chin. J. Nat. Med..

[22] Jing N., Wang M., Gao M., Zhong Z., Ma Y., Wei A. (2021). Color sensory characteristics, nutritional components and antioxidant capacity of *Zanthoxylum bungeanum* Maxim. as affected by different drying methods. Ind. Crops Prod..

[23] Chen Q., Lu X., Guo X., Guo Q., Li D. (2017). Metabolomics characterization of two *Apocynaceae* plants, *Catharanthus roseus* and Vinca minor, using GC-MS and LC-MS methods in combination. Molecules.

[24] Perez de Souza L., Alseekh S., Naake T., Fernie A. (2019). Mass spectrometry-based untargeted plant metabolomics. Curr. Protoc. Plant Biol..

[25] Tsugawa H., Cajka T., Kind T., Ma Y., Higgins B., Ikeda K., Kanazawa M., VanderGheynst J., Fiehn O., Arita M. (2015). MS-DIAL: Data-independent MS/MS deconvolution for comprehensive metabolome analysis. Nat. Methods.

[26] Moro T.M.A., Celegatti C.M., Pereira A.P.A., Lopes A.S., Barbin D.F., Pastore G.M., Clerici M.T.P.S. (2018). Use of burdock root flour as a prebiotic ingredient in cookies. LWT-Food Sci. Technol..

[27] Rahman M.S. (2019). Water activity and glass transition of foods. Reference Module in Food Science.

[28] Zhang W., Liang X. (2019). Headspace gas chromatography-mass spectrometry for volatile components analysis in *Ipomoea Cairica* (L.) sweet leaves: Natural deep eutectic solvents as green extraction and dilution matrix. Foods.

[29] Xu S., Errabelli R., Feener D.H., Noble K., Attygalle A.B. (2019). Identification of alkylpyrazines by gas chromatography mass spectrometry (GC-MS). J. Chromatogr. A.

[30] Jena S., Ray A., Sahoo A., Panda P.C., Nayak S. (2020). Deeper insight into the volatile profile of essential oil of two Curcuma species and their antioxidant and antimicrobial activities. Ind. Crops Prod..

[31] Mo X., Peng X., Liang X., Fang S., Xie H., Chen J., Meng Y. (2021). Development of antifungal gelatin-based nanocomposite films functionalized with natamycin-loaded zein/casein nanoparticles. Food Hydrocoll..

[32] Lou Z., Liu Y., Hong Y., Song X., Wang H., Ai L. (2013). Anti-biofilm activities and chemical composition of essential oil from burdock leaf. Food Sci. Technol. Res..

[33] Dong W., Hu R., Long Y., Li H., Zhang Y., Zhu K., Chu Z. (2019). Comparative evaluation of the volatile profiles and taste properties of roasted coffee beans as affected by drying method and detected by electronic nose, electronic tongue, and HS-SPME-GC-MS. Food Chem..

[34] An K., Zhao D., Wang Z., Wu J., Xu Y., Xiao G. (2016). Comparison of different drying methods on Chinese ginger (Zingiber officinale Roscoe): Changes in volatiles, chemical profile, antioxidant properties, and microstructure. Food Chem..

[35] Li Q.Q., Wang G., Huang F., Banda M., Reed E. (2010). Antineoplastic effect of beta-elemene on prostate cancer cells and other types of solid tumour cells. J. Pharm. Pharmacol..

[36] Tyagi A.K., Prasad S., Yuan W., Li S., Aggarwal B.B. (2015). Identification of a novel compound (beta-sesquiphellandrene) from turmeric (*Curcuma longa*) with anticancer potential: Comparison with curcumin. Investig. New Drugs.

[37] Sanchez-Fernandez R.E., Diaz D., Duarte G., Lappe-Oliveras P., Sanchez S., Macias-Rubalcava M.L. (2016). Antifungal volatile organic compounds from the endophyte *Nodulisporium* sp. strain GS4d2II1a: A qualitative change in the intraspecific and interspecific interactions with *Pythium aphanidermatum*. Microbial. Ecol..

[38] Clerck C., Maso S.D., Parisi O., Dresen F., Zhiri A., Jijakli M.H. (2020). Screening of antifungal and antibacterial activity of 90 commercial essential oils against 10 pathogens of agronomical importance. Foods.

[39] Dudareva N., Klempien A., Muhlemann J.K., Kaplan I. (2013). Biosynthesis, function and metabolic engineering of plant volatile organic compounds. New Phytol..

[40] Mohammadi X., Deng Y., Matinfar G., Singh A., Mandal R., Pratap-Singh A. (2020). Impact of three different dehydration methods on nutritional values and sensory quality of dried broccoli, oranges, and carrots. Foods.

[41] Ma Y., Wu X., Zhang Q., Giovanni V., Meng X. (2018). Key composition optimization of meat processed protein source by vacuum freeze-drying technology. Saudi J. Biol. Sci..

[42] Petricevic S., Marusic Radovcic N., Lukic K., Listes E., Medic H. (2018). Differentiation of dry-cured hams from different processing methods by means of volatile compounds, physico-chemical and sensory analysis. Meat Sci..

[43] Lorenzo J.M., Carballo J., Franco D. (2013). Effect of the inclusion of chestnut in the finishing diet on volatile compounds of dry-cured ham from celta pig breed. J. Integr. Agric..

[44] Gonçalves G.M.S., Barros P.P., de Silva G.H., Fedes G.R. (2019). The essential oil of *Curcuma longa* rhizomes as an antimicrobial and its composition by gas chromatography/mass spectrometry. Rev. Ciênc. Méd..

[45] Liu Y., Luo M., Liu F., Feng X., Ibrahim S.A., Cheng L., Huang W. (2020). Effects of freeze drying and hot-air drying on the physicochemical properties and bioactivities of polysaccharides from Lentinula edodes. Int. J. Biol. Macromol..

[46] Zhang J., Cao J., Pei Z., Wei P., Xiang D., Cao X., Shen X., Li C. (2019). Volatile flavour components and the mechanisms underlying their production in golden pompano (*Trachinotus blochii*) fillets subjected to different drying methods: A comparative study using an electronic nose, an electronic tongue and SDE-GC-MS. Food Res. Int..

[47] Demarchi S.M., Torrez Irigoyen R.M., Giner S.A. (2018). Vacuum drying of rosehip leathers: Modelling of coupled moisture content and temperature curves as a function of time with simultaneous time-varying ascorbic acid retention. J. Food Eng..

