# Supplementary material for: Effect of Drying Methods on Volatile Compounds of Burdock (Arctium lappa L.) Root Tea as Revealed by Gas Chromatography Mass Spectrometry-Based Metabolomics
Source: Foods. 2021 Apr 15;10(4):868. doi: 10.3390/foods10040868 (PMC8071549; doi:10.3390/foods10040868)
Supplement: Supplementary file 1 [file foods-10-00868-s001.zip › foods-1146286-supplementary.pdf]

foods

MDPI

Effect of Drying Methods on Volatile Compounds of Burdock (*Arctium lappa* L.) Root Tea as Revealed by Gas Chromatography Mass Spectrometry-Based Metabolomics

Junjie Xia [1,†], Zili Guo [1,†], Sheng Fang [2], Jinping Gu [1] and Xianrui Liang [1,*]

[1] Collaborative Innovation Center of Yangtze River Delta Region Green Pharmaceuticals, College of Pharmaceutical Sciences, Zhejiang University of Technology, Hangzhou 310014, China; xjj0718@163.com (J.X.); guozili@zjut.edu.cn (Z.G.); jinpinggu@foxmail.com (J.G.)
[2] School of Food Science and Biotechnology, Zhejiang Gongshang University, Xuezheng Street No. 18, Hangzhou 310018, China; fszjgsu@163.com
* Correspondence: liangxrvicky@zjut.edu.cn; Tel.: +86-5718-832-0420
† These two authors contributed equally to the work.

Table S1. The main 31 volatile components identified based on VIP > 1 and *p*-value < 0.05 between sun light drying (SD) and natural drying (ND).

ID	RT	Compound	VIP	*P*-value
4	1.911	Chloromethane	5.00	1.15×10^{-6}
13	3.115	*Unknown 1*	2.52	2.43×10^{-7}
14	3.116	Formic acid	1.66	9.06×10^{-8}
17	3.439	Propanal	1.29	2.83×10^{-3}
19	3.625	*Unknown 2*	1.20	8.35×10^{-3}
20	3.653	Dimethyl sulfide	7.17	4.88×10^{-4}
22	3.846	Isopropyl Alcohol	1.76	2.01×10^{-7}
26	4.595	*Unknown 3*	1.94	2.05×10^{-8}
28	4.998	2-Butanone	4.93	1.95×10^{-5}
34	6.219	*Unknown 4*	1.57	1.59×10^{-4}
44	8.36	3-Methylbutanal	6.96	9.22×10^{-5}
47	8.622	2-Methylbutanal	7.02	6.13×10^{-4}
49	9.164	Acetic acid	3.42	5.79×10^{-3}
50	9.273	2-Ethylfuran	3.08	1.89×10^{-4}
53	9.775	Pentanal	1.38	2.14×10^{-2}
78	12.48	*Unknown 5*	1.95	7.83×10^{-3}
85	13.61	Methylpyrazine	1.16	8.75×10^{-4}
95	14.48	Furfural	4.06	2.36×10^{-6}
109	15.82	2,5-Dimethylpyrazine	2.47	1.62×10^{-3}
119	16.38	1-(2-Furanyl)-ethanone	3.71	3.50×10^{-6}
122	16.72	*Unknown 6*	4.00	1.30×10^{-2}
137	17.77	5-Methyl-2-furancarboxaldehyde	1.30	5.90×10^{-6}
172	20.11	*Unknown 7*	2.15	1.88×10^{-2}
177	20.36	1-(1H-pyrrol-2-yl)-ethanone	1.09	6.37×10^{-4}
189	21.41	2-Methoxy-3-(2-methylpropyl)-pyrazine	1.71	1.28×10^{-6}
268	24.38	β-Elemene	1.63	7.45×10^{-3}
293	25.25	1-Methyl-4-(6-methylhept-5-en-2-yl) benzene	2.56	6.23×10^{-7}
295	25.32	*Unknown 8*	1.20	1.70×10^{-4}
306	25.61	β-Selinene	1.62	4.73×10^{-6}
307	25.67	α-Selinene	1.85	7.88×10^{-6}
336	26.83	*Unknown 9*	1.34	5.59×10^{-5}

Table S2. The main 29 volatile components identified based on VIP > 1 and *p*-value < 0.05 between vacuum freeze drying (VFD) and natural drying (ND).

ID	RT	Compound	VIP	*P*-value
4	1.911	Chloromethane	3.78	4.96×10^{-10}
13	3.115	*Unknown 1*	3.97	3.36×10^{-11}
14	3.116	Formic acid	2.55	5.71×10^{-11}
18	3.557	*Unknown 2*	1.65	6.33×10^{-5}
19	3.625	Unknown	1.74	2.74×10^{-6}
20	3.653	Dimethyl sulfide	9.58	2.58×10^{-7}
26	4.595	*Unknown 3*	1.13	1.68×10^{-8}
28	4.998	2-Butanone	4.49	1.01×10^{-6}
34	6.219	*Unknown 4*	1.59	8.63×10^{-6}
44	8.36	3-Methylbutanal	5.45	3.97×10^{-5}
47	8.622	2-Methylbutanal	7.18	4.82×10^{-6}
50	9.273	2-Ethylfuran	3.25	5.66×10^{-6}
53	9.775	Pentanal	1.31	2.00×10^{-3}
79	12.893	Hexanal	5.10	3.23×10^{-5}
103	15.411	2-Heptanone	1.01	1.27×10^{-4}
119	16.382	1-(2-Furanyl)-ethanone	1.85	6.28×10^{-5}
126	17.267	2-Pentylfuran	4.41	1.22×10^{-5}
162	19.472	Benzeneacetaldehyde	1.36	1.38×10^{-3}
177	20.356	1-(1H-pyrrol-2-yl)-ethanone	1.41	2.73×10^{-8}
189	21.41	2-Methoxy-3-(2-methylpropyl)-pyrazine	1.69	6.30×10^{-10}
203	21.977	*Unknown 5*	1.22	2.44×10^{-5}
281	24.881	*Unknown 6*	2.19	7.92×10^{-6}
284	24.975	1-Pentadecene	1.20	1.32×10^{-5}
293	25.251	1-(1,5-Dimethyl-4-hexenyl)-4-methylbenzene	1.59	1.66×10^{-4}
336	26.828	*Unknown 7*	4.09	4.77×10^{-13}
346	27.194	Heptadeca-1,8,11-triene	1.61	3.91×10^{-5}
348	27.333	Heptadeca-1,8,11,14-tetraene	4.52	3.39×10^{-5}
376	28.903	*Unknown 8*	1.10	3.88×10^{-4}
382	29.377	*Unknown 9*	1.83	5.33×10^{-6}

Table S3. The main 27 volatile components identified based on VIP > 1 and *p*-value < 0.05 between hot air drying at 50 °C (HD50) and natural drying (ND).

ID	RT	Compound	VIP	*P*-value
4	1.911	Chloromethane	5.98	1.12×10^{-10}
13	3.115	*Unknown 1*	3.35	2.55×10^{-7}
14	3.116	Formic acid	2.14	3.67×10^{-7}
17	3.439	Propanal	1.93	9.20×10^{-4}
26	4.595	*Unknown 2*	1.73	9.97×10^{-8}
28	4.998	2-Butanone	3.84	1.15×10^{-2}
34	6.219	*Unknown 3*	2.11	6.33×10^{-5}
47	8.622	2-Methylbutanal	6.64	1.86×10^{-2}
50	9.273	2-Ethylfuran	3.83	2.84×10^{-4}
53	9.775	Pentanal	1.68	1.87×10^{-2}
64	11.043	1-Chloropentane	1.54	1.21×10^{-2}
66	11.19	*Unknown 4*	1.04	5.11×10^{-4}
85	13.608	Methylpyrazine	1.25	6.31×10^{-3}
95	14.477	Furfural	5.07	5.92×10^{-6}

103	15.411	2-Heptanone	1.10	8.58×10^{-3}
109	15.818	2,5-Dimethylpyrazine	2.22	4.67×10^{-2}
119	16.382	1-(2-Furanyl)-ethanone	5.23	1.02×10^{-6}
122	16.722	*Unknown 5*	2.37	3.44×10^{-2}
137	17.768	5-Methyl-2-furancarboxaldehyde	1.63	9.36×10^{-6}
184	21.194	*Unknown 6*	1.08	1.49×10^{-6}
189	21.41	2-Methoxy-3-(2-methylpropyl)-pyrazine	1.79	3.00×10^{-6}
293	25.251	1-Methyl-4-(6-methylhept-5-en-2-yl) benzene	2.25	4.23×10^{-3}
303	25.547	*Unknown 7*	1.12	1.26×10^{-4}
306	25.613	β-Selinene	1.09	7.15×10^{-4}
307	25.67	α-Selinene	1.29	7.39×10^{-4}
348	27.333	(Heptadeca-1,8,11,14-tetraene	4.30	4.7×10^{-2}
350	27.418	*Unknown 8*	1.01	2.32×10^{-2}

Table S4. The main 21 volatile components identified based on VIP > 1 and p-value < 0.05 between hot air drying at 60 °C (HD60) and natural drying (ND).

ID	RT	Compound	VIP	*P*-value
4	1.911	Chloromethane	5.24	2.18×10^{-9}
13	3.115	*Unknown 1*	5.03	3.90×10^{-8}
14	3.116	Formic acid	3.22	2.99×10^{-8}
17	3.439	Propanal	1.76	4.77×10^{-4}
20	3.653	Dimethyl sulfide	11.66	7.03×10^{-7}
22	3.846	Isopropyl alcohol	1.91	6.13×10^{-5}
26	4.595	*Unknown 2*	1.55	2.38×10^{-6}
34	6.219	*Unknown 3*	1.87	6.59×10^{-5}
44	8.36	3-Methylbutanal	4.75	2.04×10^{-2}
47	8.622	2-Methylbutanal	5.22	4.03×10^{-2}
49	9.164	Acetic acid	3.74	4.60×10^{-2}
50	9.273	2-Ethylfuran	3.24	5.60×10^{-4}
55	9.996	*Unknown 4*	1.11	4.88×10^{-3}
64	11.043	1-Chloropentane	1.19	3.00×10^{-4}
66	11.19	*Unknown 5*	1.17	2.15×10^{-5}
67	11.311	*Unknown 6*	1.06	6.31×10^{-4}
78	12.481	*Unknown 7*	1.39	4.30×10^{-2}
97	14.666	*Unknown 8*	1.06	7.03×10^{-3}
122	16.722	*Unknown 9*	2.35	1.74×10^{-2}
284	24.975	1-Pentadecene	1.06	1.32×10^{-2}
336	26.828	*Unknown 10*	1.28	7.76×10^{-5}

Table S5. The main 25 volatile components identified based on VIP > 1 and p-value < 0.05 between hot air drying at 70 °C (HD70) and natural drying (ND).

ID	RT	Compound	VIP	*P*-value
13	3.115	*Unknown 1*	4.12	7.49×10^{-6}
14	3.116	Formic acid	2.63	7.95×10^{-6}
22	3.846	Isopropyl alcohol	2.83	1.18×10^{-4}
34	6.219	*Unknown 2*	2.54	9.75×10^{-6}
50	9.273	2-Ethylfuran	4.88	1.99×10^{-5}
55	9.996	*Unknown 3*	1.51	7.18×10^{-4}
79	12.893	Hexanal	8.17	4.04×10^{-5}
97	14.666	*Unknown 4*	1.32	7.18×10^{-3}

99	15.081	*Unknown 5*	1.03	4.59×10^{-2}
103	15.411	2-Heptanone	1.53	3.04×10^{-4}
109	15.818	2,5-Dimethylpyrazine	3.53	3.26×10^{-3}
122	16.722	*Unknown 6*	2.48	4.41×10^{-2}
126	17.267	2-Pentylfuran	6.24	1.43×10^{-4}
134	17.542	Benzaldehyde	1.22	1.04×10^{-5}
143	17.948	*Unknown 7*	1.28	1.58×10^{-2}
160	19.403	Pyrazinamide	1.40	5.52×10^{-6}
163	19.509	*Unknown 8*	1.38	3.10×10^{-4}
174	20.176	Nonanal	1.12	6.28×10^{-6}
177	20.356	1-(1H-pyrrol-2-yl)-ethanone	1.31	2.20×10^{-4}
203	21.977	*Unknown 9*	1.13	1.94×10^{-2}
205	22.1	Decanal	1.11	8.68×10^{-6}
284	24.975	1-Pentadecene	1.34	5.86×10^{-3}
290	25.172	γ-Curcumene	1.08	1.96×10^{-3}
351	27.521	*Unknown 10*	1.67	1.68×10^{-8}
376	28.903	*Unknown 11*	1.54	4.56×10^{-5}

Table S6. The main 30 volatile components identified based on VIP > 1 and *p*-value < 0.05 between hot air drying at 80 °C (HD80) and natural drying (ND).

ID	RT	Compound	VIP	*P*-value
4	1.911	Chloromethane	6.47	5.57×10^{-9}
7	2.318	Methanethiol	1.01	2.65×10^{-4}
13	3.115	*Unknown 1*	4.17	3.88×10^{-9}
14	3.116	Formic acid	2.67	4.33×10^{-9}
17	3.439	Propanal	1.88	3.91×10^{-5}
18	3.557	*Unknown 2*	1.42	2.48×10^{-3}
19	3.625	*Unknown 3*	1.57	7.26×10^{-4}
20	3.653	Dimethyl sulfide	8.12	2.03×10^{-4}
26	4.595	*Unknown 4*	1.83	6.02×10^{-9}
28	4.998	2-Butanone	4.38	3.26×10^{-4}
34	6.219	*Unknown 5*	1.87	3.01×10^{-5}
44	8.36	3-Methylbutanal	5.15	1.24×10^{-2}
47	8.622	2-Methylbutanal	5.80	1.83×10^{-2}
49	9.164	Acetic acid	3.79	1.07×10^{-2}
50	9.273	2-Ethylfuran	3.69	3.61×10^{-5}
53	9.775	Pentanal	1.72	1.18×10^{-2}
54	9.974	2,3-Pentanedione	1.61	1.52×10^{-2}
64	11.066	1-Chloropentane	1.34	1.04×10^{-5}
66	11.19	*Unknown 6*	1.34	5.51×10^{-7}
95	14.477	Furfural	3.55	1.38×10^{-4}
97	14.666	*Unknown 7*	1.09	5.15×10^{-3}
119	16.382	1-(2-Furanyl)-ethanone	2.72	4.00×10^{-4}
137	17.768	5-Methyl-2-furancarboxaldehyde	1.07	1.64×10^{-4}
152	19.074	Unknown 8	1.09	9.59×10^{-6}
168	19.83	3,5-Octadien-2-one	1.03	1.61×10^{-5}
202	21.973	*Unknown 9*	1.26	5.69×10^{-4}
203	21.977	*Unknown 10*	2.07	1.54×10^{-2}
268	24.375	β-Elemene	1.03	1.29×10^{-3}

307	25.67	α-Selinene	1.15	1.13×10^{-3}
369	28.444	β-Eudesmol	1.71	3.70×10^{-7}

Table S7. The main 32 volatile components identified based on VIP > 1 and *p*-value < 0.05 between vacuum drying at 50 °C (VD50) and natural drying (ND).

ID	RT	Compound	VIP	*P*-value
4	1.911	Chloromethane	6.60	5.28×10^{-6}
13	3.115	*Unknown 1*	2.75	2.70×10^{-4}
14	3.116	Formic acid	1.77	2.36×10^{-4}
17	3.439	Propanal	1.46	4.30×10^{-2}
26	4.595	*Unknown 2*	1.69	7.39×10^{-5}
28	4.998	2-Butanone	4.32	1.27×10^{-2}
34	6.219	*Unknown 3*	1.87	3.49×10^{-5}
36	6.327	*Unknown 4*	1.65	1.21×10^{-2}
47	8.622	2-Methylbutanal	7.68	1.30×10^{-2}
49	9.164	Acetic acid	4.97	8.17×10^{-3}
50	9.273	2-Ethylfuran	3.44	2.04×10^{-4}
64	11.066	1-Chloropentane	1.11	2.35×10^{-3}
66	11.19	*Unknown 5*	1.52	1.14×10^{-4}
79	12.893	Hexanal	4.74	6.87×10^{-3}
85	13.608	Methylpyrazine	1.01	1.76×10^{-2}
95	14.477	Furfural	4.35	7.64×10^{-4}
97	14.666	*Unknown 6*	1.02	9.35×10^{-3}
103	15.411	2-Heptanone	1.01	6.47×10^{-3}
109	15.818	2,5-Dimethylpyrazine	2.43	7.06×10^{-3}
119	16.382	1-(2-Furanyl)-ethanone	4.72	3.86×10^{-4}
137	17.768	5-Methyl-2-furancarboxaldehyde	1.59	6.34×10^{-4}
177	20.356	1-(1H-pyrrol-2-yl)-ethanone	1.33	5.32×10^{-3}
189	21.41	2-Methoxy-3-(2-methylpropyl)-pyrazine	1.40	1.02×10^{-3}
203	21.977	*Unknown 7*	1.09	5.26×10^{-3}
226	23.031	*Unknown 8*	1.05	2.87×10^{-2}
268	24.375	β-Elemene	1.08	1.67×10^{-2}
296	25.361	*Unknown 9*	2.19	2.22×10^{-5}
306	25.613	β-Selinene	1.05	3.94×10^{-3}
307	25.67	α-Selinene	1.10	9.83×10^{-3}
336	26.828	*Unknown 10*	1.03	2.06×10^{-3}
369	28.444	β-Eudesmol	2.48	1.90×10^{-3}
371	28.517	*Unknown 11*	1.24	2.71×10^{-3}

Table S8. The main 21 volatile components identified based on VIP > 1 and *p*-value < 0.05 between vacuum drying at 60 °C (VD60) and natural drying (ND).

ID	RT	Compound	VIP	*P*-value
13	3.115	*Unknown 1*	4.67	5.34×10^{-8}
14	3.116	Formic acid	3.00	6.50×10^{-8}
18	3.557	*Unknown 2*	1.69	9.98×10^{-4}
19	3.625	*Unknown 3*	1.82	1.10×10^{-3}
20	3.653	Dimethyl sulfide	10.11	2.85×10^{-5}
24	4.194	Methylene chloride	1.44	2.52×10^{-7}
34	6.219	*Unknown 4*	2.03	1.71×10^{-5}
49	9.164	Acetic acid	4.82	1.29×10^{-2}

50	9.273	2-Ethylfuran	3.81	5.36×10^{-5}
66	11.19	*Unknown 5*	1.49	1.96×10^{-6}
79	12.893	Hexanal	5.49	1.15×10^{-3}
95	14.477	Furfural	2.04	4.01×10^{-2}
103	15.411	2-Heptanone	1.21	6.88×10^{-4}
126	17.267	2-Pentylfuran	4.35	2.85×10^{-3}
133	17.542	Benzaldehyde	1.21	3.96×10^{-2}
155	19.193	Phenol	2.91	5.13×10^{-6}
162	19.472	Benzeneacetaldehyde	1.39	4.83×10^{-2}
203	21.977	*Unknown 6*	1.36	9.71×10^{-6}
221	22.761	*Unknown 7*	1.31	8.92×10^{-7}
274	24.616	*Unknown 8*	1.08	2.07×10^{-6}
376	28.903	*Unknown 9*	1.28	8.29×10^{-5}

Table S9. The main 15 volatile components identified based on VIP > 1 and p-value < 0.05 between vacuum drying at 70 °C (VD70) and natural drying (ND).

ID	RT	Compound	VIP	*P*-value
13	3.115	*Unknown 1*	4.34	3.89×10^{-6}
14	3.116	Formic acid	2.79	4.08×10^{-6}
24	4.194	Methylene chloride	1.37	2.11×10^{-6}
34	6.219	*Unknown 2*	1.86	7.62×10^{-6}
50	9.273	2-Ethylfuran	3.70	8.35×10^{-6}
55	9.996	*Unknown 3*	1.03	8.21×10^{-3}
79	12.893	Hexanal	5.52	1.62×10^{-4}
97	14.666	*Unknown 4*	1.04	3.63×10^{-3}
103	15.411	2-Heptanone	1.19	1.37×10^{-4}
109	15.818	2,5-Dimethylpyrazine	1.84	1.66×10^{-3}
122	16.722	*Unknown 5*	1.08	7.22×10^{-3}
126	17.267	2-Pentylfuran	4.59	1.72×10^{-4}
153	19.181	Phenol	1.07	1.05×10^{-4}
203	21.977	*Unknown 6*	1.39	1.88×10^{-2}
284	24.975	1-Pentadecene	1.02	1.13×10^{-2}

Table S10. The main 24 volatile components identified based on VIP > 1 and p-value < 0.05 between vacuum drying at 80 °C (VD80) and natural drying (ND).

ID	RT	Compound	VIP	*P*-value
4	1.911	Chloromethane	1.95	3.36×10^{-5}
13	3.115	*Unknown 1*	4.41	0.19×10^{-2}
14	3.116	Formic acid	2.83	9.51×10^{-5}
18	3.557	*Unknown 2*	1.58	1.79×10^{-3}
19	3.625	*Unknown 3*	1.67	7.77×10^{-4}
20	3.653	Dimethyl sulfide	8.49	3.80×10^{-4}
28	4.998	2-Butanone	4.36	1.60×10^{-2}
34	6.219	*Unknown 4*	1.99	9.93×10^{-6}
50	9.273	2-Ethylfuran	3.93	1.29×10^{-5}
79	12.893	Hexanal	5.30	1.89×10^{-3}
97	14.666	*Unknown 5*	1.11	4.04×10^{-3}
103	15.411	2-Heptanone	1.25	2.44×10^{-4}
126	17.267	2-Pentylfuran	4.50	1.37×10^{-3}
160	19.403	Pyrazinamide	1.10	1.26×10^{-2}
168	19.83	3,5-Octadien-2-one	1.03	1.78×10^{-3}

177	20.356	1-(1H-pyrrol-2-yl)-ethanone	1.11	4.48×10^{-2}
202	21.973	*Unknown 6*	1.14	1.96×10^{-3}
203	21.977	*Unknown 7*	1.81	2.90×10^{-3}
284	24.975	1-Pentadecene	1.24	2.25×10^{-3}
293	25.251	1-Methyl-4-(6-methylhept-5-en-2-yl) benzene	2.61	3.03×10^{-6}
296	25.361	*Unknown 8*	1.03	2.47×10^{-4}
346	27.194	Heptadeca-1,8,11-triene	1.49	2.61×10^{-2}
354	27.577	*Unknown 9*	1.51	3.76×10^{-2}
376	28.903	*Unknown 10*	1.01	3.91×10^{-2}

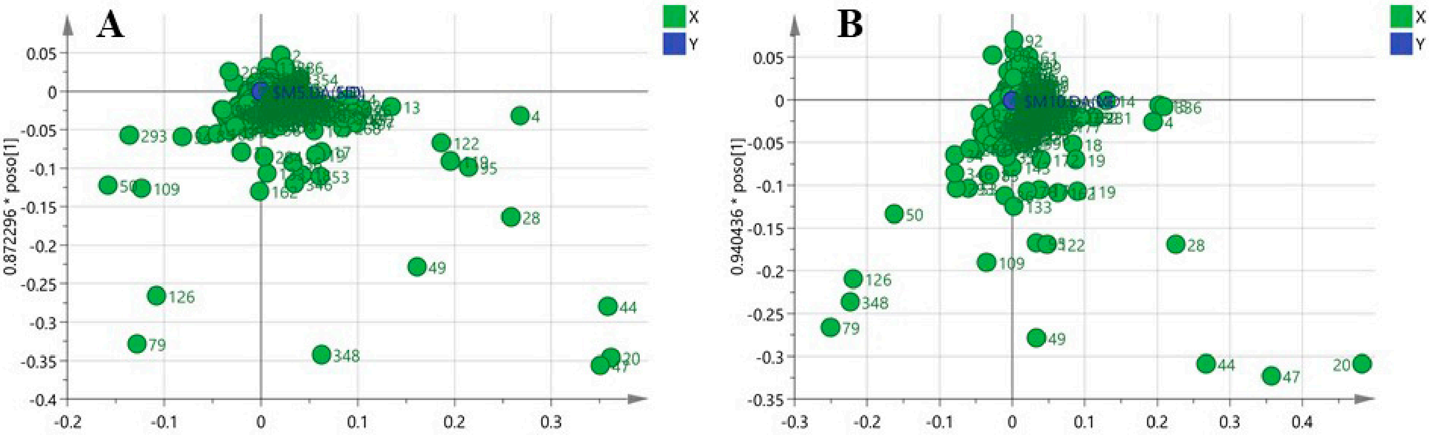

Figure S1. The loading plots: (**A**) between natural drying (ND) and sun light drying (SD), (**B**) between ND and vacuum freeze drying (VFD).

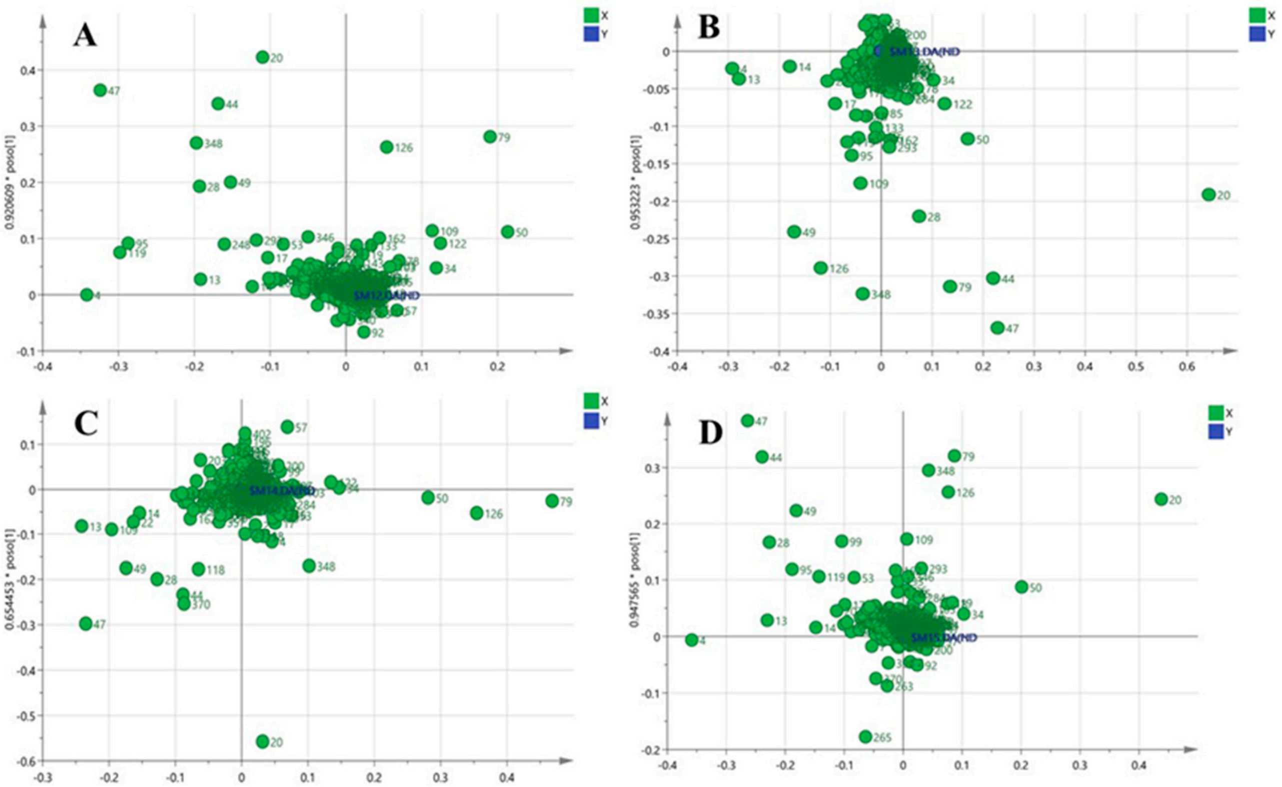

Figure S2. The loading plots for comparisons between the different temperatures of hot air drying (HD) and natural drying (ND): (**A**) for HD50 and ND, (**B**) for HD60 and ND, (**C**) for HD70 and ND, (**D**) for HD80 and ND.

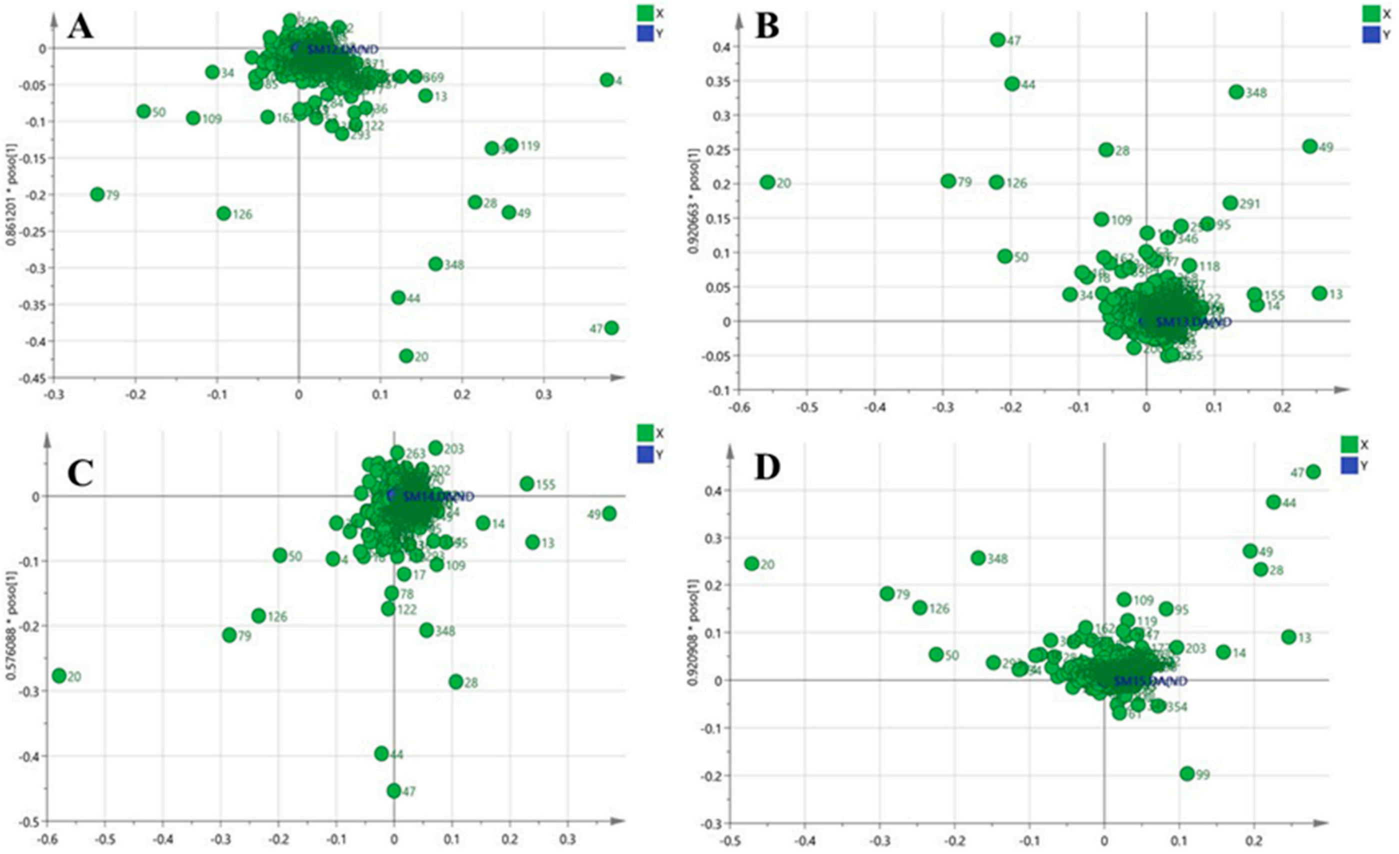

Figure S3. The loading plots for comparisons between the different temperatures of vacuum drying (VD) and natural drying (ND): (**A**) for VD50 and ND, (**B**) for VD60 and ND, (**C**) for VD70 and ND, (**D**) for VD80 and ND.
